# Horizontal Transfer of Virulence Factors by Pathogenic Enterobacteria to Marine Saprotrophic Bacteria during Co-Cultivation in Biofilm

**DOI:** 10.3390/biotech11020017

**Published:** 2022-05-24

**Authors:** Alena I. Eskova, Boris G. Andryukov, Anatoli A. Yakovlev, Alexandra V. Kim, Anna L. Ponomareva, Vera S. Obuhova

**Affiliations:** 1 Somov Research Institute of Epidemiology and Microbiology by Federal Service for Surveillance on Consumer Rights Protection and Human Wellbeing, 690087 Vladivostok, Russia; andrukov_bg@mail.ru (B.G.A.); yakovlev-epid@yandex.ru (A.A.Y.); geomicrob@mail.ru (V.S.O.); 2 Il’ichev Pacific Oceanological Institute, Far Eastern Branch of the Russian Academy of Sciences, 690022 Vladivostok, Russia; ponomareva.al@poi.dvo.ru; 3 School of Medicine, Far Eastern Federal University (FEFU), 690091 Vladivostok, Russia; kim-sandra@mail.ru; 4 Federal Scientific Center of the East Asia Terrestrial Biodiversity of the Far Eastern Branch of the Russian Academy of Sciences, 690022 Vladivostok, Russia

**Keywords:** *Yersinia pseudotuberculosis*, *Listeria monocytogenes*, co-cultivation of bacteria, marine saprotrophic bacteria, pathogenicity factors, horizontal transfer of genes, islands of pathogenicity, interspecies interaction, polycultural biofilm

## Abstract

Environmental problems associated with marine pollution and climate warming create favorable conditions for the penetration and survival of pathogenic bacteria in marine ecosystems. These microorganisms have interspecific competitive interactions with marine bacteria. Co-culture, as an important research strategy that mimics the natural environment of bacteria, can activate silent genes or clusters through interspecies interactions. The authors used modern biotechnology of co-cultivation to dynamically study intercellular interactions between different taxa of bacteria—pathogenic enterobacteria *Yersinia pseudotuberculosis* and *Listeria monocytogenes* and saprotrophic marine bacteria *Bacillus* sp. and *Pseudomonas japonica* isolated in summer from the coastal waters of the recreational areas of the Sea of Japan. The results of the experiments showed that during the formation of polycultural biofilms, horizontal transfer of genes encoding some pathogenicity factors from *Y. pseudotuberculosis* and *L. monocytogenes* to marine saprotrophic bacteria with different secretion systems is possible. It was previously thought that this was largely prevented by the type VI secretion system (T6SS) found in marine saprotrophic bacteria. The authors showed for the first time the ability of marine bacteria *Bacillus* sp. and *P. japonica* to biofilm formation with pathogenic enterobacteria *Y. pseudotuberculosis* and *L. monocytogenes*, saprophytic bacteria with type III secretion system (T3SS). For the first time, a marine saprotrophic strain of *Bacillus* sp. Revealed manifestations of hyaluronidase, proteolytic and hemolytic activity after cultivation in a polycultural biofilm with listeria. Saprotrophic marine bacteria that have acquired virulence factors from pathogenic enterobacteria, including antibiotic resistance genes, could potentially play a role in altering the biological properties of other members of the marine microbial community. In addition, given the possible interdomain nature of intercellular gene translocation, acquired virulence factors can be transferred to marine unicellular and multicellular eukaryotes. The results obtained contribute to the paradigm of the epidemiological significance and potential danger of anthropogenic pollution of marine ecosystems, which creates serious problems for public health and the development of marine culture as an important area of economic activity in coastal regions.

## 1. Introduction

Bacteria co-cultivation technologies are widely used in microbial ecology for the dynamic study of intercellular interactions between different bacterial taxons, evolution and adaptation to changing environmental conditions. The possibility of horizontal transfer of not only antibiotic resistance genes but also those encoding certain pathogenicity factors in natural environments has attracted wide attention in recent years [1,2].

Recent studies have shown that horizontal gene transfer (HGT) and biofilm formation are interrelated processes in mixed cultures of bacteria. It has been established that the ability of microorganisms to form biofilms is one of the mechanisms of their adaptation to the effects of adverse environmental conditions. This mediates the preservation and maintenance of the population of pathogenic bacteria, including when it enters the marine environment with various domestic, industrial and other effluents [1,3].

In recent years, it has become increasingly clear that under natural conditions, biofilms are more often formed not by one but by several types of bacteria [4]. This increases the fundamental and practical significance of studying polycultural biofilms [5]. There is more and more evidence that the presence of microorganisms in a particular natural environment is determined not only by its conditions but also by the presence of control by other microorganisms. The role of signaling molecules produced by microorganisms acting as intra- or interspecies regulators of microbial interaction has been established. In addition, the significance of mobile genetic elements (MGE) in the intercellular HGT encoding of some bacterial functions was determined [2,3,6].

It has been established that microbial interactions, including horizontal genetic exchange, signaling, and metabolite exchange, occur between microorganisms in biofilm communities [1,7,8,9]. It has been found that within a biofilm, bacteria of different taxa form a single genetic system in the form of plasmids and other MGE that carry a behavioral code for members of the biofilm and determine their trophic, energy, and other connections between themselves and the outside world [2,4,9,10]. Microbial interaction in these consortia leads to complex relationships leading not only to growth suppression, but also to absorption, transfer/acquisition of DNA elements, leading to the emergence of new metabolic properties, properties in community members [1,3,4,6].

However, experimental studies of this plan are few, especially when pathogenic enterobacteria and marine bacteria are co-cultivated. In this regard, studies seem to be very significant, allowing us to establish: to what extent the marine environment is a “cemetery” of microorganisms and to what time it is a “bridgehead” for the formation of epidemic variants of pathogenic bacteria [7,10].

*Yersinia pseudotuberculosis* is a widespread pathogen of sapronous infections. Due to the two-phase paradigm of existence (in the body of warm-blooded organisms and the environment), the *Listeria monocytogenes* can lead parasitic and saprophytic lifestyles [8,11].

In the course of our previous studies [12], the possibility of preserving the pathogenic properties of *L. monocytogenes* and *Y. pseudotuberculosis* in marine ecosystems was experimentally shown, but also the ability of marine saprotrophs to modulate the growth activity of these enterobacteria [12,13]. In addition, an assumption was made about the possibility of HPG coding for pathogenicity factors from enteropathogens to marine bacteria (including factors that ensure the interaction of pathogens with the epithelium, persistence, and secretion of bacterial modulins and toxins).

On the other hand, in recent years it has been established that marine bacteria have a type 6 secretion system (T6SS), which is actively used by them in interspecies competition. The labeling of bacteria containing T6SS implies their predatory behavior is associated not only with growth suppression, but also with the absorption of competing bacteria [3].

This work aimed to evaluate the possibility of transferring pathogenic characteristics from pathogenic enterobacteria (*Y. pseudotuberculosis* and *L. monocytogenes*) to marine saprotrophic bacteria after their co-cultivation.

## 2. Materials and Methods

### 2.1. Bacterial Isolates Identification and Characterization

We used the certified strains *Y. pseudotuberculosis* No. 3515 and *Listeria monocytogenes* No. 870 from the Research Institute of Epidemiology and Microbiology collection, as well as two strains of marine saprotrophic bacteria *Bacillus* sp. and *Pseudomonas japonica* isolated from the waters of the Golden Horn Bay coastal area of the Sea of Japan. Seawater samples were stored in the dark at a temperature of 2–3 °C throughout the entire preanalytical period [14].

Morphological characterization color, shape, transparency and margin were examined and recorded as colony morphological characteristics according to Matsui (2003) [6]. Microscopic features were recorded for all isolates via the Gram stain protocol. Bacteria were identified by classical methods based on morphological, cultural, physiological and biochemical properties and by molecular genetic methods based on the determination of the 16 S rRNA gene sequence. The 16S rRNA gene was amplified using the universal primers 27F (5′-AGAGTTTGATCCTGGCTCAG-3′) and 1492R (5′-GGTTACCTTGTTACGACTT-3′). To determine the genera of the studied bacteria, differentiating tests were carried out by Bergey’s determinant [15]. To confirm the taxonomic status of the strains, strips with API 20 biochemical tests from BioMerieux (Craponne, France) were used. The results obtained were interpreted using an electronic database (https://www.biomerieux.fr/, accessed on 4 May 2022).

Water samples were taken in summer at a temperature of 20 °C from a depth of 10–15 cm at a distance of 0.5 km from the shore with a sterile syringe V = 20 mL in three duplicates. The requirements of GOST are to carry out microbiological studies (GOST is a national standard that complies with international water quality standard ISO 19458:2006—Sampling for microbiological analysis (revision 2020)). Water samples were sown by the Drygalski method [16].

Cultivation of marine bacteria was carried out on solid nutrient medium SMM (supplemented liquid minimal media) [11], *L. monocytogenes* on DDSL medium (differential diagnostic medium for *Listeria*) [14], and *Y. pseudotuberculosis* on CIN agar [17] at room temperature (20 °C). When assessing the ability of all studied bacteria to biofilm formation, 24-well plates were used. Cultivation at a concentration of 10^3^ CFU/mL was carried out in a liquid medium for marine microorganisms (SMM) [14] at a temperature of 20 °C for 5 days. For the preparation of samples for PCR, bacterial cells were harvested while in the exponential growth phase, centrifuged, washed in 1 M NaCl, resuspended in a TE buffer (10 mM Tris, 1 mM EDTA, pH 8), and diluted to an optical density of 1.0 at 600 nm.

Molecular fingerprinting as a confirmatory identification technique was applied to extract total bacterial genomic DNA. The 16S rRNA gene *L. monocytogenes* was amplified using the universal primers prs1 and prs2 to determine the *prs* gene encoding a genus-specific general metabolism protein, phosphoribosyl pyrophosphate synthase, the nucleotide sequence of which was obtained from the ListiList database (http://genolist.pasteur.fr/ListiList/ (accessed on 17 April 2022), containing the genome sequence of the *L. monocytogenes* EGDe strain [14]. Identification of isolated cultures of *Listeria* to the species *L. monocytogenes* was carried out using the PCR test system. Thermal cycler conditions were designed as 94 °C for 5 min, 3 cycles at 94 °C for 45 s, 57 °C for 30 s, 72 °C for 120 s; 3 cycles at 94 °C for 45 s, 56 °C for 30 s, 72 °C for 120 s; 3 cycles at 94 °C for 45 s, 55 °C for 30 s, 72 °C for 120 s; 26 cycles at 94 °C for 45 s, 53 °C for 30 s, 72 °C for 120 s; and a final step at 72 °C for 5 min [14].

The YersiniaBase analytical platform (http://yersinia.um.edu.my, accessed on 08 April 2022) was used to analyze *Y. pseudotuberculosis* genomic data [18]. The 16S rRNA gene was amplified using the universal primers 27F (5′-AGAGTTTGATCCTGGCTCAG-3′) and 1492R (5′-GGTTACCTTGTTACGACTT-3′). Identification of isolated cultures of *Yersinia* to the species *Y. pseudotuberculosis* was carried out using the PCR test system. Real-time PCR was performed on a real-time system with the following conditions: 95 °C for 2 min followed by 45 cycles of 95 °C for 15 s and 64 °C for 60 s. Distilled water was always included as a negative control. A primer concentration that generates a sigmoidal shape of the amplification curve with a relatively lower Ct value compared to other primer concentrations is considered an optimized concentration.

### 2.2. Determination of the Ability for Mono- and Polybiofilm Formation by the Studied Strains

The detection of mono- and multicultural biofilms of microorganisms was carried out on the abiotic surface using the spectrophotometric method [19]. Optical density (OD) was measured over 5 days of incubation in 96-well plates (observing 3-fold replication) on a plate reader (Labsystems iEMS Reader MF, BioRad) at ƛ = 540 nm). Wells with media without bacteria served as controls. As previously proposed by us [12], following the OD values, all the studied microorganisms were divided into two groups depending on their ability for biofilm formation: weak biofilm formers (OD < 0.4) and strong biofilm formers (OD > 0.4).

To determine the dynamics of bacterial reproduction in a monoculture and an association, the number of microorganisms in the planktonic and biofilm phases was considered. The growth of biofilms formed by each strain was studied on Petri dishes [20]. Accounting for the number was determined by the number of colony-forming units in 1 mL (CFU/mL). For further experiments, strains grown on elective media after co-cultivation in the biofilm were used. Cell morphology and culture purity were checked using an Axioskop 40 light microscope, Carl Zeiss, Germany [16,21]. Five percent blood agar was used to study hemolytic activity [22]. Petri dishes with blood agar were seeded with an 18-h culture of microorganisms. The plates were incubated at 22 ± 1 °C for 24–48 h. After that, the results were recorded. With a positive reaction, the appearance of a greenish-brown halo around the colonies or the formation of a transparent zone of hemolysis was noted [22].

### 2.3. Study of the Pathogenicity Factors of the Studied Strains

**Figure 1:**
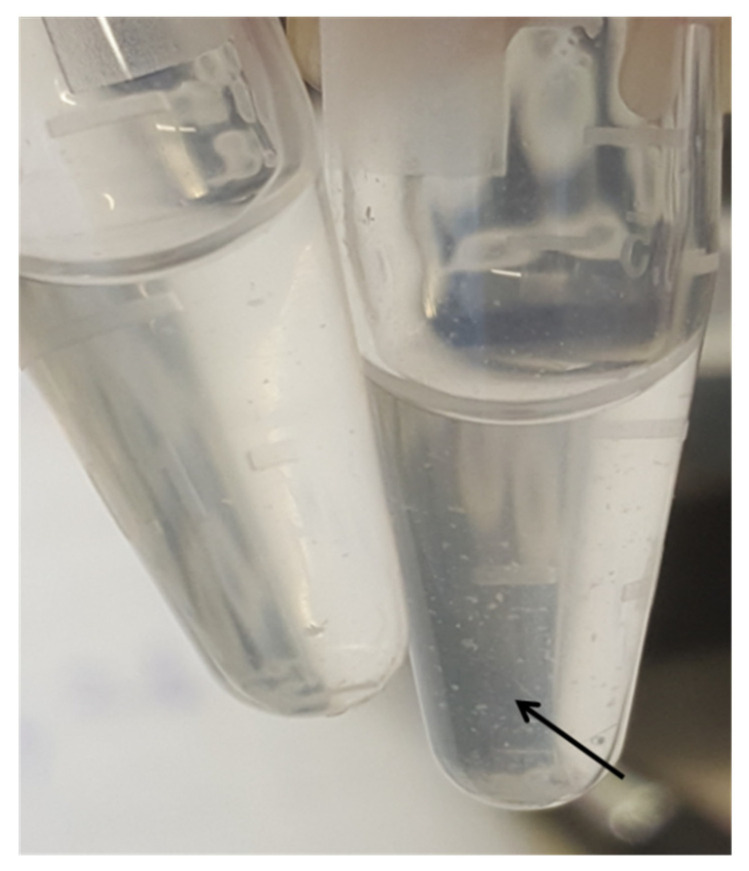
Determination of hyaluronidase activity: on the left—control, on the right—the arrow shows clots, indicating a positive reaction.

Hyaluronidase activity was studied in sterile Eppendorf’s; 0.3 mL of hyaluronic acid in a working titer was poured into them, and 0.05 mL of 2 billion suspensions of bacteria were added and put in a thermostat (36 ± 1 °C) for 15 min, and then in the refrigerator for 5 min. Next, 5 drops of 15% acetic acid were instilled into the wells, causing hyaluronic acid to coagulate, and the results were taken into account. The flocculation was taken into account after 5 min [23,24] (Figure 1).

Determination of plasma-coagulase activity was examined in sterile Eppendorf tubes; 0.3 mL of hyaluronic acid in a working titer was poured into them, and 0.05 mL of 2 billion suspensions of bacteria were added. Then, they were placed in a thermostat (36 ± 1 °C) for 15 min and then in a refrigerator for 5 min. A positive reaction was noted in the absence of a coagulated clot [23].

The study of bacterial adhesion of marine bacteria was carried out according to the Brillis method in 24-well plates [23]. To account for the adhesive properties of bacteria, human erythrocytes O (I) of the Rh (+) blood group were used since these cells contain a substance glycophorin on their surface, which is identical to the glycocalyx of epithelial cells on the surface of which there are receptors for adhesins of microorganisms [23,25,26]. A suspension of bacteria was prepared at a concentration of 10^9^ cells/mL. The bacteria were incubated on a plate at 37 ± 1 °C.

**Figure 2:**
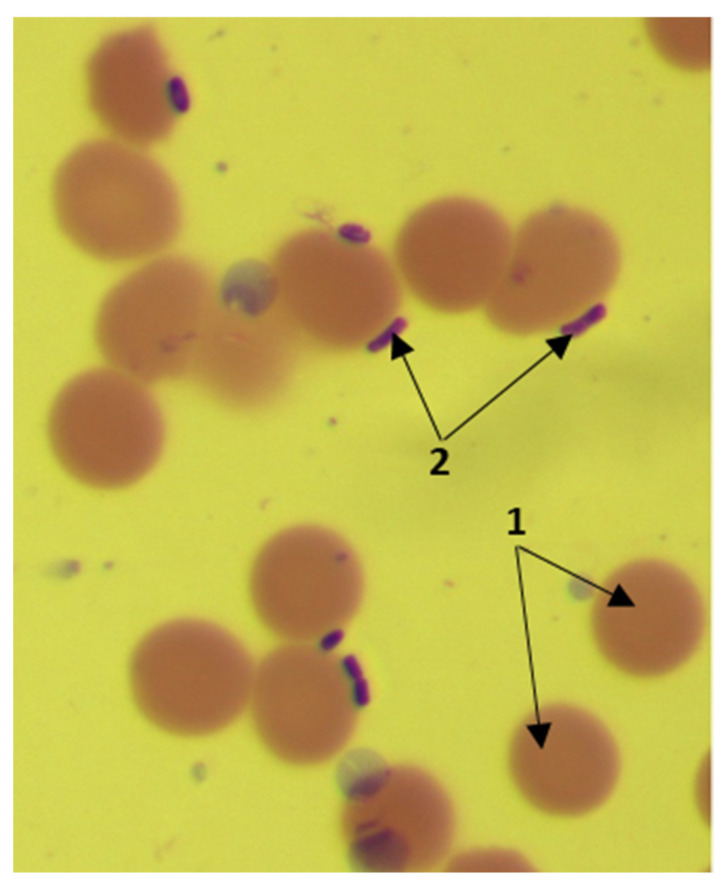
When studying the adhesive properties on slides stained according to Romanowsky–Giemsa staining, erythrocytes are stained dark pink and bacterial cells in purple. Designations: 1—erythrocytes; 2—bacterial cells.

Adhesion was studied under an Axioskop 40 light microscope (Carl Zeiss, Germany). When assessing the adhesive properties of microorganisms, the following indicators were used: the average adhesion index (AAI), the coefficient of participation of cells in the adhesive process K and the index adhesiveness of microorganism (IAM). AAI was used to use to determine the adhesion level of microorganisms to erythrocyte, which is the average number of microorganisms attached to the surface of a single red blood cell. Counting was carried out on 100 cells looking through the entire glass slide. Microorganisms were considered non-adhesive when IAM was less than 1.75, low-adhesive—from 1.76 to 2.5—medium-adhesive—from 2.51 to 4.0—and highly adhesive when IAM was above 4.0. When studying the adhesive properties on slides stained according to Romanowsky–Giemsa staining, erythrocytes were stained dark pink, and bacterial cells were in purple (Figure 2).

### 2.4. Statistical Processing of Results

Statistical analysis of the study results was carried out to identify the reliability of trends, patterns and relationships using quantitative data on changes in the properties of marine bacteria as a result of co-cultivation with enterobacteria. The null hypothesis is the absence of the effect of co-cultivation; an alternative theory is the formation of pathogenic properties in the studied strains of marine bacteria due to co-cultivation with enterobacteria. Statistical processing of the results was carried out in Microsoft Office Excel 2007. When the *p*-value < 0.05, the probability of error in rejecting the null hypothesis is less than 5%.

## 3. Research Results and Discussion

### 3.1. Selection of Strains for the Experiment, Their Identification

In previous studies [12], we proved the ability of *L. monocytogenes* 870 and *Y. pseudotuberculosis* 3515 to form both monocultural and polycultural biofilms with marine saprotrophs.

*Bacillus* sp. is a Gram-positive and while *P. japonica* is Gram-negative bacteria. Different types of bacteria (with different cell wall compositions and not closely related) were chosen for the experiment. Possible ways of transferring genes responsible for pathogenic properties and virulence (islands of pathogenicity, etc.) from pathogenic microorganisms to their related species are known in the literature. Changes in pathogenic properties after co-cultivation of pathogenic bacteria with saprotrophic microorganisms with cell walls of different structures were not found in the literature. In this regard, we decided to experimentally test the possibility of transferring pathogenic properties between such bacteria, so we used saprotrophic microorganisms of different species.

We determined that the strains of marine microorganisms used in our work isolated from the waters of the Golden Horn Bay had the ability to biofilm formation (the average OD values and the standard deviation between the series of measurements in terms of the ability to biofilm formation were 0.530 ± 0.03 arb. units and 0.560 ± 0.02 arb. units, respectively). It allowed us to use them in our experiment. The strain was identified as *Bacillus* sp., and the strain was assigned to *P. japonica*. The assessment of the degree of formation of polycultural biofilms and the reproduction dynamics was carried out according to proven methods [27,28].

### 3.2. The Study of Some Properties of Pathogenicity of the Studied Strains

#### 3.2.1. Determination of Hemolytic Activity

To determine the hemolytic activity, bacterial strains grown on each experiment day from a polycultural biofilm were used. Strains from monocultural biofilms served as a control for comparison. To differentiate marine saprotrophs from opportunistic bacteria, inoculation was carried out on differential diagnostic media indicated in the methods, and the individual formed colonies were microscopically examined.

**Figure 3:**
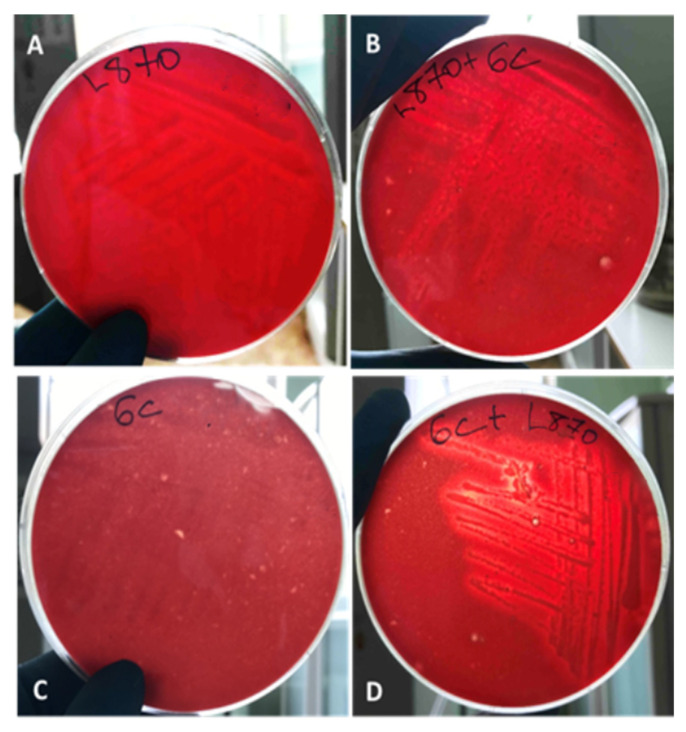
Growth on blood agar 48–72-h biofilms of *Bacillus* sp. after co-cultivation with *L. monocytogenes*: (**A**)—the absence of a transparent zone of hemolysis around the colonies of *L. monocytogenes* (monobiofilm); (**B**)—partial lysis around the colonies of *L. monocytogenes*, sown after co-cultivation with *Bacillus* sp.; (**C**)—absence of a transparent zone of hemolysis around the colonies of the *Bacillus* sp. strain (monobiofilm); (**D**)—the appearance of a transparent zone of hemolysis around the colonies of the saprotroph *Bacillus* sp. after co-cultivation in biofilm with *L. monocytogenes*.

On Petri dishes with blood agar for the primary separation, all the studied microorganisms were seeded after cultivation in mono- or multicultural biofilm from each experiment day. After 24–48 h on the plates, the appearance of a transparent zone of hemolysis or the formation of a greenish-brown halo around the colonies was noted. In our experiment, erythrocyte lysis was characteristic of *L. monocytogenes* 870 from the biofilm with *Bacillus* sp. from the third day of cultivation in a joint biofilm (Figure 3B). *Listeria* itself in monoculture also did not have a visible zone of hemolysis on the third day of cultivation (Figure 3A).

According to the literature, *Listeria* is characterized by β-hemolysis on blood agar [27,28,29]. Furthermore, cultivation with a saprotrophic marine strain was unfavorable for Listeria (for example, competition for nutrients, etc.) conditions. The strain of *Bacillus* sp. did not show any hemolytic properties in the monoculture (Figure 3C). However, for *L. monocytogenes* 870, a decrease in the ability to lyse erythrocytes was noted when co-cultivated in a biofilm with *Bacillus* sp.

#### 3.2.2. Determination of Plasma-Coagulase Activity

The next stage of the experiment was the determination of plasma-coagulase activity. According to the literature data [30], the plasma coagulase enzyme is present in Gram-negative bacteria, including *Pseudomonas*. However, in our experiment, the enzyme plasma coagulase was not detected in *Pseudomonas* either in mono- or polycultural biofilms. *Yersinia* showed plasma-coagulase activity after co-cultivation with *Bacillus* sp. In monoculture, *Yersinia* did not have plasma-coagulase activity. The plasma-coagulase activity was observed in both Gram-positive bacteria (*L. monocytogenes* 870 and *Bacillus* sp.) after co-cultivation on the third day.

#### 3.2.3. Determination of Hyaluronidase Activity

The enzyme hyaluronidase can cleave mucopolysaccharides, which are part of marine plants and, therefore, are found in the marine environment [31]. Similar enzymes can be active in microorganisms in clean and polluted water bodies. The enzyme hyaluronidase, which destroys hyaluronic acid, was noted in Listeria both in a monofilm and after cultivation with Bacillus sp. on the third and fifth days. Hyaluronidase activity in Listeria was also recorded after co-cultivation with P. japonica. According to the literature, hyaluronidase activity is characteristic of Listeria (stable sign) [32].

The appearance of hyaluronidase activity in the saprotrophic strain of *Bacillus* sp. (3 days after co-cultivation with *Listeria* in biofilm). In monoculture, hyaluronidase activity in this microorganism was not observed. It once again confirms the possibility of the appearance of pathogenic properties in the saprotrophic strain of *Bacillus* sp. just after co-cultivation with *Listeria*.

#### 3.2.4. Evaluation of the Adhesive Properties of the Studied Bacteria

The assessment of the adhesive properties of the studied bacteria made it possible to establish that, according to the IAM indicators, the strain of *Bacillus* sp. was characterized as low adhesive. The IAM value was 1.8 ± 0.2 (*p* < 0.05). After co-cultivation in a biofilm with *L. monocytogenes* 870, on the third day, the IAM was 8.3 ± 0.11, and on the fifth day, it was 8.0 ± 0.15, which significantly exceeds its value in the monofilm. According to the results of our study, the *P. japonica* strain turned out to be non-adhesive throughout the day of the experiment. The adhesiveness of *Listeria* in the monofilm was moderate (IAM 3.75 ± 0.05, at *p* < 0.05), but after cultivation with *Bacillus* sp. indicators of IAM exceeded 4.1 ± 0.13. So, on the third and fifth days of co-cultivation, IAM values were 5.12 ± 0.21 and 7.14 ± 0.12, respectively. In a consortium with *P. japonica*, the listeria adhesion index was 6.02 ± 0.16 on the third day of cultivation and 6.61 ± 0.22 on the fifth.

The adhesion index of *Yersinia* in a monofilm was average (2.63 ± 0.05, *p* < 0.05). After cultivation in a mixed biofilm with *Bacillus* sp. strain became low-adhesive (IAM 1.81 ± 0.11). According to the literature, bacteria of the genus *Yersinia*, in particular *Y. pseudotuberculosis*, can lose or acquire pathogenicity islands in adaptation to unfavorable conditions [26,33,34]. Thus, cultivation with the *P. japonica* strain increased the adhesion index in *Yersinia* to an average value (3.12 ± 0.12) on the fifth day of cultivation [22,35,36,37]. Thus, cultivation with the *P. japonica* strain led to an increase in the adhesion index in *Yersinia* to an average value (3.12 ± 0.12) on the fifth day of cultivation.

At the same time, during the experiment, a decrease in the abundance of the saprotrophic strain *P. japonica* was noted on the third and subsequent days after co-cultivation in a biofilm with the pathogens used in work.

Coculture, as an important research strategy that mimics the natural environment of bacteria, can activate silent genes or clusters through interspecies interactions [38,39]. Unlike standard laboratory growing conditions, co-cultivation is not only an effective method for studying the biosynthesis of various secondary metabolites and enzymes of microorganisms but is important for revealing the mechanisms of interspecific competitive and symbiotic relationships between enteropathogens and marine bacteria, as well as new gene functions [40,41,42].

The results obtained are a clear illustration of the possibility of acquiring pathogenicity factors by marine saprotrophic bacteria after co-cultivation with pathogenic enterobacteria. These bacteria, due to pollution of the oceans and warming of coastal waters, inhabit marine ecosystems and interact with marine bacteria [38,39]. These interactions are mediated by a wide range of mechanisms and very often involve the secretion of various molecules from bacterial cells. The results of the experiments were obtained using routine microbiological studies, but they are quite convincing. Much has been written in recent years about the type VI secretion system (T6SS) found in marine bacteria as an effective weapon of interbacterial competition [43,44,45].

Over the past two decades, there has been a shift in microbiological research towards the use of systems approaches to study the interactions between diverse organisms and their communities in an ecological context.

Our results show that the presence of T6SS in marine bacteria is not an obstacle to the horizontal transfer of virulence factors from pathogenic enterobacteria that have entered marine ecosystems. As a possible mechanism, first of all, it is necessary to assume the horizontal transfer of mobile genetic elements encoding the secretion of new proteins, enzymes that change the biological properties of marine bacteria and mediate the acquisition of virulence factors by them.

Intercellular possible strategies for genetic exchange include plasmid-mediated conjugation, bacteriophage transduction, and transformation via bacterial uptake of extracellular DNA.

Here, we will focus on this aspect of the acquisition of new biochemical properties by marine bacteria. Future studies on the implications of co-culture of enteropathogens and marine bacteria using whole-genome sequencing (WGS) molecular techniques will shed light on the transgenic mechanism.

## 4. Conclusions

Thus, during the experiment, we found:

1. The strain of *Bacillus* sp. after co-cultivation with *Listeria* acquired hemolytic, plasma-coagulase and hyaluronidase activities. When this saprophyte was co-cultivated with *Yersinia*, these pathogenic properties did not appear in it.
2. *Yersinia,* after co-cultivation with *Bacillus* sp., appeared in plasma coagulase activity. The adhesiveness index of *Yersinia* after cultivation in a mixed biofilm with *Bacillus* sp. has decreased, while cultivation with the *P. japonica* 3P9 strain, on the contrary, led to an increase in this index in *Yersinia*.
3. For *L. monocytogenes* 870, a partial ability to lyse erythrocytes and the appearance of hyaluronidase activity was noted when co-cultivated in a biofilm with *Bacillus* sp. The adhesiveness of *Listeria* in a monofilm was 3.75 ± 0.05, but after cultivation with *Bacillus* sp., it reached 4.1 ± 0.13. The *P. japonica* strain did not show potential pathogenic properties both in the monofilm and after co-cultivation with pathogens.
4. The results of the experiments showed the possibility of transferring virulent properties from enterobacteria to saprotrophic marine bacteria *Bacillus* sp., as well as a decrease in the severity of some pathogenicity factors in the tested pathogens (and a reduction of *Yersinia* adhesion after cultivation in a mixed biofilm with *Bacillus* sp.). A change in the pathogenic properties of *Listeria* and *Yersinia* was revealed—the appearance of the ability to lyse erythrocytes, plasma-coagulase activity and an increase in the adhesiveness of *Listeria* after cultivation with *Bacillus* sp. and in *Yersinia* after co-cultivation with *P. japonica.*

Marine bacteria make up the largest part of the biomass in the oceans and play a key role not only in the microbial community, but also in the functioning of the entire ecosystem. Environmental problems associated with sea pollution and global warming create favorable conditions for pathogenic bacteria to enter and remain viable in marine ecosystems. In addition to the epidemiological threat, these microorganisms have competitive interactions with marine bacteria [35].

Our experimental studies have shown that the co-cultivation of pathogenic enterobacteria and nonpathogenic saprotrophic marine bacteria in a biofilm can lead to the horizontal transfer of genes encoding pathogenicity factors between non-closely related species of prokaryotes with different secretion systems. Previously, it was believed [36] that this is largely prevented by the use of the type VI secretion system (T6SS) in marine saprotrophic bacteria, which is one of the least studied competitive strategies among heterotrophic bacteria living in marine ecosystems.

The authors have shown for the first time the ability of marine bacteria *Bacillus* sp. and *P. japonica* for biofilm formation with pathogenic enterobacteria *Y. pseudotuberculosis* and *L. monocytogenes*, saprophytic bacteria with type III secretion systems (T3SS). For the first time, using traditional microbiological methods, not only the possibility of co-cultivation of pathogenic and marine bacteria with different secretion systems in the biofilm, but also the possibility of intercellular horizontal transfer of some virulence properties from pathogenic enterobacteria to marine saprotrophic bacteria, was shown. Being part of a biofilm formed by various strains of microorganisms, bacteria can contribute to better adaptation and survival of pathogenic bacteria in the marine environment and influence the formation of pathogenic properties in saprophytic marine bacteria.

To further elucidate the mechanism of horizontal transfer of pathogenicity factors to marine prokaryotes, it is necessary to use additional conditions and methodological approaches, for example, third-generation genome-wide sequestration technology and genetic editing based on CRISPR-Cas9, which will help confirm the fact of horizontal gene transfer and identify the involved mobile genetic elements. This understanding will allow us to confirm the importance of mobile elements in the evolution of marine microorganisms and a deeper understanding of the mechanisms of interaction, and to draw attention to the growing importance of the environmental and epidemiological problems of pollution of the World Ocean, which increases with climate warming on the planet.

## Data Availability

Not applicable.

## References

[1] Madsen J.S., Burmølle M., Hansen L.H., Sørensen S.J. (2012). The interconnection between biofilm formation and horizontal gene transfer. FEMS Immunol. Med. Microbiol..

[2] Andryukov B.G., Besednova N.N., Zaporozhets T.S. (2022). Mobile Genetic Elements of Prokaryotes and Their Role in the Formation of Antibiotic Resistance in Pathogenic Bacteria. Antibiot. Chemother..

[3] Coyne M.J., Roelofs K.G., Comstock L.E. (2016). Type VI secretion systems of human gut Bacteroidales segregate into three genetic architectures, two of which are contained on mobile genetic elements. BMC Genom..

[4] Browne K., Kuppusamy R., Chen R., Willcox M.D.P., Walsh W.R., Black D.S., Kumar N. (2022). Bioinspired Polydopamine Coatings Facilitate Attachment of Antimicrobial Peptidomimetics with Broad-Spectrum Antibacterial Activity. Int. J. Mol. Sci..

[5] Kittredge H.A., Dougherty K.M., Evans S.E. (2022). Dead but Not Forgotten: How Extracellular DNA, Moisture, and Space Modulate the Horizontal Transfer of Extracellular Antibiotic Resistance Genes in Soil. Appl. Environ. Microbiol..

[6] Matsui K., Ishii N., Kawabata Z. (2003). Microbial interactions affecting the natural transformation of Bacillus subtilis in a model aquatic ecosystem. FEMS Microbiol. Ecol..

[7] Burton A.T., Kearns D.B. (2020). The Large pBS32/pLS32 Plasmid of Ancestral Bacillus subtilis. J. Bacteriol..

[8] Sun D. (2018). Pull in and Push Out: Mechanisms of Horizontal Gene Transfer in Bacteria. Front. Microbiol..

[9] Hasegawa H., Suzuki E., Maeda S. (2018). Horizontal Plasmid Transfer by Transformation in Escherichia coli: Environmental Factors and Possible Mechanisms. Front. Microbiol..

[10] Goers L., Freemont P., Polizzi K.M. (2014). Co-culture systems and technologies: Taking synthetic biology to the next level. J. R. Soc. Interface.

[11] Joint I., Mühling M., Querellou J. (2010). Culturing marine bacteria—An essential prerequisite for biodiscovery. Microb. Biotechnol..

[12] Eskova A.I., Yakovlev A.A., Kim A.V. (2021). Modeling of Interspecific Interaction of Yersinia pseudotuberculosis and Listeria monocytogenes in a Mixed-Species Biofilms with Microorganisms Isolated from Coastal Waters of the Sea of Japan. Bull. Exp. Biol. Med..

[13] Yakovlev A.A., Rakov A.V., Pozdeeva E.S. (2020). Significance of interspecies and intraspecies interactions of microorganisms as a sub-organism level in the hierarchy of the epidemic process. Epidemiol. Infect. Dis..

[14] Jones G.S., D’Orazio S.E. (2013). *Listeria monocytogenes*: Cultivation and Laboratory Maintenance. Curr. Protoc. Microbiol..

[15] Garrity G.M., Brenner D.J., Krieg N.R., Staley J.R., Manual B.S., Garrity G., Brenner D.J., Krieg N.R., Staley J.R. (2005). 2. The Proteobacteria, Part B: The Gammaproteobacteria. Bergey’s Manual of Systematic Bacteriology.

[16] Fukushima H., Gomyoda M. (1986). Growth of Yersinia pseudotuberculosis and Yersinia enterocolitica biotype 3B serotype O3 inhibited on cefsulodin-Irgasan-novobiocin agar. J. Clin. Microbiol..

[17] Speare L., Woo M., Dunn A.K., Septer A.N. (2022). A Putative Lipoprotein Mediates Cell-Cell Contact for Type VI Secretion System-Dependent Killing of Specific Competitors. mBio.

[18] Landrigan P.J., Stegeman J.J., Fleming L.E., Allemand D., Anderson D.M., Backer L.C., Brucker-Davis F., Chevalier N., Corra L., Czerucka D. (2020). Human Health and Ocean Pollution. Ann. Glob. Health.

[19] Tan S.Y., Dutta A., Jakubovics N.S., Ang M.Y., Siow C.C., Mutha N.V., Heydari H., Wee W.Y., Wong G.J., Choo S.W. (2015). YersiniaBase: A genomic resource and analysis platform for comparative analysis of Yersinia. BMC Bioinform..

[20] Konieczny M., Rhein P., Czaczyk K., Białas W., Juzwa W. (2021). Imaging Flow Cytometry to Study Biofilm-Associated Microbial Aggregates. Molecules.

[21] Deng Y., Xu H., Su Y., Liu S., Xu L., Guo Z., Wu J., Cheng C., Feng J. (2019). Horizontal gene transfer contributes to virulence and antibiotic resistance of Vibrio harveyi 345 based on complete genome sequence analysis. BMC Genom..

[22] Lasa I., Del Pozo J.L., Leiva J., Penadés J.R. (2005). Bacterial biofilms and infection. An. Sist. Sanit. Navar..

[23] Brilis V.I., Brilene T.A., Lentsner K.P., Lentsner A.A. (1986). Metodika izucheniya adgezivnogo processa mikroorganizmov. Lab. Delo.

[24] Tolker-Nielsen T. (2014). Pseudomonas aeruginosa biofilm infections: From molecular biofilm biology to new treatment possibilities. APMIS Suppl..

[25] Giaouris E., Heir E., Desvaux M., Hébraud M., Møretrø T., Langsrud S., Doulgeraki A., Nychas G.-J., Kačániová M., Czaczyk K. (2015). Intra- and inter-species interactions within biofilms of important foodborne bacterial pathogens. Front. Microbiol..

[26] Bukharin O.V. (2013). Symbiotic Interactions of Microorganisms During Infenction. Mikrobiol. Epidemiol. Immunobiol..

[27] Terekhova V.E., Sosnin V.A., Buzoleva L.S., Shakirov R.B. (2010). Distribution of the bacteria Listeria monocytogenes in the western part of the Sea of Okhotsk. Oceanology.

[28] Pushkareva V.I., Ermolaeva S.A. (2018). Experimental Evidences On A Crop Plant Role In Epidemiology Of Sapronotic (Soil-Borne) Bacterial Infections. J. Microbiol. Epidemiol. Immunobiol..

[29] Falardeau J., Walji K., Haure M., Fong K., Taylor G., Ma Y., Smukler S., Wang S., Taylor G. (2018). ANative bacterial communities and Listeria monocytogenes survival in soils collected from the Lower Mainland of British Columbia, Canada. Can. J. Microbiol..

[30] Huang Z., Mo S., Yan L., Wei X., Huang Y., Zhang L., Zhang S., Liu J., Xiao Q., Lin H. (2021). A Simple Culture Method Enhances the Recovery of Culturable Actinobacteria From Coastal Sediments. Front. Microbiol..

[31] Cleland C.E. (2007). Epistemological issues in the study of microbial life: Alternative terran biospheres?. Stud. Hist. Philos. Sci. Part C Stud. Hist. Philos. Biol. Biomed. Sci..

[32] Yunusova I.O., Yakovlev A.A. (2021). The role of metabolites in the interspecific interaction of bacteria (review). Sanit. Dr..

[33] Costerton J.W., Stewart P.S., Greenberg E.P. (1999). Bacterial Biofilms: A Common Cause of Persistent Infections. Science.

[34] Juhas M., van der Meer J.R., Gaillard M., Harding R.M., Hood D.W., Crook D.W. (2009). Genomic islands: Tools of bacterial horizontal gene transfer and evolution. FEMS Microbiol. Rev..

[35] Norman A., Hansen L.H., Sørensen S.J. (2009). Conjugative plasmids: Vessels of the communal gene pool. Philos. Trans. R. Soc. B Biol. Sci..

[36] Schmidt H., Hensel M. (2004). Pathogenicity Islands in BacterialPathogenesis. Clin. Microbiol. Rev..

[37] Carniel E. (2001). The Yersinia high-pathogenicity island: An iron-uptake island. Microbes Infect..

[38] Kurpas M., Osek J., Moura A., Leclercq A., Lecuit M., Wieczorek K. (2020). Genomic Characterization of Listeria monocytogenes Isolated From Ready-to-Eat Meat and Meat Processing Environments in Poland. Front. Microbiol..

[39] Jung H. (2020). Hyaluronidase: An overview of its properties, applications, and side effects. Arch. Plast. Surg..

[40] Yu G., Sun Y., Han H., Yan X., Wang Y., Ge X., Qiao B., Tan L. (2021). Coculture, An Efficient Biotechnology for Mining the Biosynthesis Potential of Macrofungi via Interspecies Interactions. Front. Microbiol..

[41] Xu X.-Y., Shen X.-T., Yuan X.-J., Zhou Y.-M., Fan H., Zhu L.-P., Du F.-Y., Sadilek M., Yang J., Qiao B. (2018). Metabolomics Investigation of an Association of Induced Features and Corresponding Fungus during the Co-culture of Trametes versicolor and Ganoderma applanatum. Front. Microbiol..

[42] Shen X.-T., Mo X.-H., Zhu L.-P., Tan L.-L., Du F.-Y., Wang Q.-W., Zhou Y.-M., Yuan X.-J., Qiao B., Yang S. (2019). Unusual and Highly Bioactive Sesterterpenes Synthesized by *Pleurotus ostreatus* during Coculture with *Trametes robiniophila* Murr. Appl. Environ. Microbiol..

[43] Salomon D., Klimko J.A., Trudgian D.C., Kinch L., Grishin N.V., Mirzaei H., Orth K. (2015). Type VI Secretion System Toxins Horizontally Shared between Marine Bacteria. PLoS Pathog..

[44] Kempnich M.W., Sison-Mangus M.P. (2020). Presence and abundance of bacteria with the Type VI secretion system in a coastal environment and in the global oceans. PLoS ONE.

[45] Gallegos-Monterrosa R., Coulthurst S.J. (2021). The ecological impact of a bacterial weapon: Microbial interactions and the Type VI secretion system. FEMS Microbiol. Rev..

